# Selective re-partnering? Mental health and life satisfaction among separated single mothers in Germany and the UK

**DOI:** 10.1177/00016993241300435

**Published:** 2025-01-08

**Authors:** Philipp Dierker, Mine Kühn, Mikko Myrskylä

**Affiliations:** Department of Social Demography, 9162 Max Planck Institute for Demographic Research, Rostock, Germany; 111628 Helsinki Institute for Demography and Population Health, University of Helsinki, Helsinki, Finland; 9162 Max Planck – 111628 University of Helsinki Center for Social Inequalities in Population Health, Rostock, Germany and Helsinki, Finland; Department of Sociology, 120694 Tilburg School of Social and Behavioral Sciences, Tilburg University, Tilburg, The Netherlands; Department of Social Demography, 9162 Max Planck Institute for Demographic Research, Rostock, Germany; 111628 Helsinki Institute for Demography and Population Health, University of Helsinki, Helsinki, Finland; 9162 Max Planck – 111628 University of Helsinki Center for Social Inequalities in Population Health, Rostock, Germany and Helsinki, Finland

**Keywords:** Lone mothers, selection, union formation, remarriage, Germany, United Kingdom

## Abstract

This study examines the potential influence of selection on the association between re-partnering and single mothers’ mental health and life satisfaction in Germany and the United Kingdom. Drawing on extensive longitudinal panel data, we analyze the trajectories of 1694 separated single mothers in Germany (SOEP) and 1070 in the UK (BHPS/UKHLS). Employing fixed effects models, we examine the outcomes before and after entry into single motherhood and compare trajectories of stably single mothers and re-partnered single mothers. In both countries, the findings weakly indicate that prior to entering single motherhood, re-partnered mothers exhibit higher levels of life satisfaction, suggesting positive selection. Increasing differences in life satisfaction after the transition into single motherhood between mothers that re-partner and stably single mothers indicate a positive association of re-partnering and life satisfaction. No evidence of mental health selection into re-partnering was found in either country, but the trajectory of re-partnered mothers in Germany shows a stronger increase than that of mothers who remain single.

## Introduction

Previous research has demonstrated that single mothers experience poorer mental and physical health and lower life satisfaction than their partnered counterparts (Baranowska-Rataj et al., 2014; Pollmann-Schult, 2018). Consequently, a new partner within the household may partially mitigate the health and well-being disadvantages associated with single motherhood (Williams and Umberson, 2004). Prior research has indicated that re-partnering has positive effects on both mental health and life satisfaction (Dierker et al., 2024; Gloor et al., 2021; Lin et al., 2019). These beneficial outcomes of re-partnering are often attributed to stress reduction resulting from shared parenting responsibilities and additional financial resources (Cooper et al., 2009; Pollmann-Schult, 2018).

Nonetheless, it is important to consider the potential influence of selection into re-partnering related to mental health and life satisfaction to gain insight into the possible reinforcement of inequalities in health and well-being. Prior research has found that socio-demographic factors, such as age and education, are selective forces in re-partnering (de Ree and Alessie, 2011; Van Winkle, 2018). In this paper, we focus on selection by mental health and life satisfaction, as both are components of subjective well-being (Diener et al., 1999), and both have been shown to be outcomes of re-partnering and other family transitions (e.g., Gloor et al., 2021; Kühn et al., 2023; Lin et al., 2019). Previous research has shown that family dynamics are drivers of inequalities in subjective well-being, aligning with theoretical approaches, such as the marital resource model and the crisis perspective (Williams and Umberson, 2004). However, these theories focus primarily on the impact of family transitions on well-being, whereas the social selection theory assumes that health and well-being factors lead to people ending up in certain family states (Carr and Springer, 2010). If potentially beneficial family transitions occur mainly among individuals who are already in better health than others, individuals in poorer health may experience a cumulative disadvantage (Dannefer, 2020). Inequalities in well-being between individuals with different family statuses would be further increased if those who are most vulnerable in terms of well-being are less likely to enter a new partnership that could improve their well-being.

From a life course perspective (Bernardi et al., 2019; Elder, 1994; Fasang et al., 2024), single motherhood is an episode in the family life stage that is often associated with reduced well-being. The assumption of health- and well-being-related selection into re-partnering aligns with Mayer's (2023) assertion that health can be a cause, a consequence, and a dimension of life course inequality. Building on the life course framework of Fasang et al. (2024), our study investigates to what extent a mother's well-being, even before she experiences a separation, shapes her subsequent family life course. Specifically, we compare the well-being trajectories of mothers who re-partnered with those of mothers who remained single, both before and after the separation, in Germany and the United Kingdom (UK). This allows us to examine how different institutional contexts, including different family policies, can change the impact of mothers’ well-being on their family life course.

Rather than identifying causal effects of re-partnering, we aim to examine the differences before the initial separation between mothers who re-partner and those who stay single, which remains underexplored in previous research. Pevalin and Ermisch (2004) examined social selection into re-partnering, but did not focus on single mothers. Among the studies focusing on single mothers, Recksiedler and Bernardi (2019) did not explicitly test health-related selection into re-partnering, while Kühn et al. (2023) focused only on the Finnish context and on antidepressant purchasing as outcome. While antidepressant purchasing is an objective indicator of the treatment of mental disorders, unlike indicators such as life satisfaction and mental health, it could also reflect help-seeking behavior.

In our study, we draw on panel data from Germany and the UK and follow mothers over a period of several decades, including during their entry into singlehood through separation, which is the most prevalent pathway into single motherhood (Kühn et al., 2023). We compare the trajectories of mental health and life satisfaction of mothers who re-partner and mothers who stay single to examine the extent to which potential differences between re-partnered and stably single mothers might be attributable to selection. Investigating this in both Germany and the UK enables us to examine whether institutional differences at the macro level, such as different family policies, change the impact of mothers’ well-being on their family life course.

## Theoretical background

There are two potential sources of well-being differences between partnered and single individuals. A partnership improves individuals’ well-being due to the increased resources and social support (causation) and people with better well-being are more attractive on the partner market and therefore more likely to be in a partnership (selection) (Carr and Springer, 2010). While the causal effects of union formation in general and of re-partnering in particular have been frequently addressed in existing research, well-being-related selection into re-partnering has rarely been investigated. However, this is especially relevant for single mothers, who represent a particularly vulnerable group with regard to well-being (Pollmann-Schult, 2018). Investigating whether single mothers with higher well-being are more likely to re-partner is important, as well-being may predict future family trajectories of separated mothers, reflecting their often complex life courses (Fasang et al., 2024; Mayer, 2023). Additionally, if well-being influences re-partnering, mothers with lower well-being may face reduced chances of re-partnering, which could set off a cycle of cumulative disadvantage, leading to further declines in well-being (Dannefer, 2020).

### Differences and similarities between mental health and life satisfaction

The measures of mental health and life satisfaction focus on different aspects of subjective well-being (Diener et al., 1999). Mental health captures emotional reactions, and thus represents the affective dimension of well-being (Ware et al., 1996). By contrast, life satisfaction is a comprehensive assessment of a person's life as a whole, and therefore represents the cognitive dimension of well-being (Diener, 1984). Examining both components can reveal whether potential selection into re-partnering based on well-being is due to cognitively evaluated well-being (life satisfaction) or to affective and emotional well-being (mental health).

### Selection into re-partnering by mental health and life satisfaction

The marriage selection hypothesis posits that individual health predicts marital transitions, with healthier individuals being more likely to enter into marriage than their less healthy counterparts (Carr and Springer, 2010). Consequently, it is likely that re-partnered mothers have better health before entering a new partnership, but evidence remains limited. Using Finnish register data, Kühn et al. (2023) found that compared to single mothers who did not re-partner, single mothers who re-partnered within four years following a separation had a lower probability of purchasing antidepressants even prior to the separation. In a British sample, Pevalin and Ermisch (2004) detected evidence of positive social selection only for re-partnering following cohabitation but not following marriage, without particularly focusing on single mothers. In a comparative analysis of six European countries, Recksiedler and Bernardi (2019) argued that the absence of significant associations between re-partnering trajectories and single mothers’ health outcomes may be attributed to the selective nature of re-partnering itself.

We hypothesize that separated single mothers who re-partner represent a positively selected group with regard to their mental health and life satisfaction, in that they have higher levels of life satisfaction and mental health before their entry into single motherhood than mothers who remain single (*Hypothesis 1*).

### Differences between Germany and the UK

The health disadvantage experienced by single mothers varies depending on the institutional context in which they reside (Nieuwenhuis et al., 2018). Consequently, the impact of re-partnering on the mental health and life satisfaction of single mothers – and potentially the degree of selectivity into re-partnering based on these well-being measures – may vary across countries with differing welfare state provisions and family policies (see Recksiedler and Bernardi, 2019). Our study examines the association between re-partnering and mental health and life satisfaction among single mothers in Germany and the UK. In both countries, the single-mother household (i.e., the child resides with the mother) represents the predominant family living arrangement following parental separation (Geisler and Kreyenfeld, 2019; Office for National Statistics, 2021).

The social welfare and family policies affecting single parents differ between both countries. Germany has higher family allowances than the UK, and the impact of family allowances on the reduction of poverty risk for single mothers is stronger in Germany (Maldonado and Nieuwenhuis, 2015). In Germany, unemployed single parents can apply for unemployment benefits, and a parent who receives no child maintenance from the other parent can apply for advance child support payments from the state (Zagel et al., 2022). In the UK, unemployed or low-income single parents can apply for Universal Credit, which includes monthly financial support for living costs and the option to apply for housing support (Aerts et al., 2022; Harkness, 2016). More comprehensive descriptions of these benefits are provided in several studies (e.g., Zagel and Hübgen, 2018; Zagel and Van Lancker, 2022).

Building on the life course framework of Fasang et al. (2024), we aim to examine whether institutional differences between Germany and the UK shape the extent to which single mothers select into re-partnering based on their well-being. Due to differences in family policies and state support, we expect UK mothers to face more financial pressure to re-partner than those in Germany. If this pressure does not vary by well-being, it suggests financial scarcity is the main driver for re-partnering in the UK. Therefore, we hypothesize that well-being-based selection into re-partnering is stronger in Germany than in the UK *(Hypothesis 2)*.

## Analytical approach

### Data

For Germany, we use data from the German Socio-Economic Panel (SOEP), a national panel study based on a representative sample of private households in Germany that has been conducted annually since 1984 (Goebel et al., 2018). For the UK, we use two datasets, starting with the British Household Panel Study (BHPS), which began in 1991 as an annual survey of a nationally representative sample of households. In 2009, this survey was transformed into the UKHLS but the datasets can be harmonized (University of Essex, 2022). We include data from the entire survey period of 1984–2020 for Germany, while for the UK, we only analyze the period from 1996–2020, as key variables of our analyses were only surveyed from 1996 onward.

We compare the trajectories of the re-partnered and the non-re-partnered single mothers before and after they entered single motherhood through separation from a male partner. Subsequently, we track both mothers who re-partnered and mothers who remained single over a period of five years. The sample comprises 2764 individuals, 730 of whom re-partnered within five years of the separation, resulting in a total of 14,496 person-years of observation. Observations later than five years after separation are censored. Mothers who stayed single until this time point but re-partnered later are defined as stably single mothers. As we hypothesize that re-partnering is selective, mothers who re-partnered shortly after censoring could distort the results, which must be considered when interpreting the findings. However, since previous research has shown that most mothers re-partner in the first five years after separation, this is unlikely to substantially bias the results (Ott et al., 2011). We exclude observations with missing values pertaining to partnership status as well as the outcome and control variables.

### Variables

#### Re-partnering

We distinguish between mothers who remained continuously single following a separation and single mothers who re-partnered. We operationalize single mothers as separated women who reside in a household with at least one biological child under age 18. Entry into single motherhood is defined as the transition that occurs when the mother's male partner leaves the household. We define a mother as re-partnered when a new male partner enters the household after a period of single motherhood and maintains a shared living arrangement with the mother and her children. Accordingly, due to this household-focused definition of single motherhood we do not consider living-apart-together (LAT) partnerships.

#### Life satisfaction and mental health

The outcomes are life satisfaction and mental health. In the German sample, participants were asked on an annual basis to rate their life satisfaction by responding to the question: “How satisfied are you with your life, all things considered?” The response options ranged from zero (completely dissatisfied) to 10 (completely satisfied). In the British sample, life satisfaction has been measured as: “Please choose the number which you feel best describes how dissatisfied or satisfied you are with the following aspects of your current situation: Your life overall.” The response scale for this item ranges from one (completely dissatisfied) to seven (completely satisfied). To enhance comparability between the two samples, we rescaled the British life satisfaction items to a range of zero to 10.

Mothers’ mental health was assessing based on the SF-12 questionnaire, which comprises 12 questions covering eight dimensions of health-related quality of life (Ware et al., 1996). The exact wording of the questions is presented in the online appendix. In the German data, information on mental health has been collected biennially since 2002, while in the British data, it has been collected annually since 2009.

#### Controls

Our models control for education, which is assessed using the ISCED classification in the German dataset, and is categorized into six distinct groups in the British dataset. We have dichotomized the education levels into “high” and “low” educational attainment. Detailed information on the coding of the education variables is in the online appendix.

Furthermore, the group of single mothers is likely to become more heterogeneous over time as it increases in size. Thus, we control for five-year calendar year dummies and five-year age dummies in all models. We have chosen these categories based on a comparison of model fit indices (presented in the online appendix).

### Method

We perform fixed effects linear regression models that estimate the trajectories of life satisfaction and mental health in the years surrounding the separation, covering both short-term (1–2 years) and long-term (3–5 years) changes. This is modeled as

$$Y_{it}=\alpha_{i}+\theta_{0}E_{0,it}+\theta_{1-2}A_{1-2,it}+\theta_{3-5}A_{3-5,it}+\beta^{\prime }X_{it}+\varepsilon_{it}$$

where $Y_{it}$ represents either life satisfaction or mental health for individual *i* at time *t*; $\alpha_{i}$ is the individual fixed effect; and X is a vector of covariates. $E_{0}$ and $A_{k}$ denote different points in time on the trajectories. Here, $E_{0}$ signifies the event of separation and $A_{k}$ captures the effects *k* years after the separation. All coefficients $\theta_{k}$ indicate effects relative to the reference point of life satisfaction or mental health 1–2 years prior to the separation.

We calculate eight unweighted models for the main analyses, stratified by outcome (mental health and life satisfaction), re-partnering status (re-partnered within five years of the separation and not re-partnered within five years of the separation), and country (Germany and the UK). This approach ensures to control for time-invariant factors within the individual groups of the respective models, while the comparison of predicted values between mothers who re-partner and mothers who remain single allows us to work out selection into re-partnering based on the differences in life satisfaction and mental health between these two groups.

We prefer sample split models over interaction terms, as we are interested in differences at all time points and especially in selection, i.e., differences in predicted values −2/-1 years before separation and in the year of separation. In fixed effects models with interaction terms, the predicted values would be fixed at one point of the time to/from separation variable, and no differences in the predictions could be recovered. Accordingly, significance of the differentials between re-partnered and non-re-partnered single mothers is obtained using bootstrapped standard errors and the coefficients of all models are presented in Table A3 in the appendix (p. 4). We acknowledge the potential selectivity of our sample (single mothers who separate and either stay single at for least five years thereafter or re-partner), and have considered this in the interpretation of our findings.

## Results

### Descriptive results

Table 1 presents the composition of our sample, which includes 1694 separated single mothers from Germany and 1070 separated single mothers from the UK. Of these single mothers, 27.2% in Germany and 22.5% in the UK had entered a new partnership within five years of the separation. Re-partnered mothers remained single for an average of 2.2 years in Germany and two years in the UK. Furthermore, in both countries about twice as many mothers had re-partnered in the two years after the separation than in three to five years thereafter.

**Table 1.** Descriptive statistics.

	Germany	UK
Re-partnered within 5 years (%)	27.2%	22.5%
Years from entry into singlehood until re- partnering (mean years)	2.2	2.0
Years from entry into singlehood until re-partnering among the re-partnered (number of individuals)		
+1/+2 years	314	149
+3/+5 years	156	79
N (individuals)	1694	1070
N (person-years)	9269	5227

*Source*: SOEP (1984–2020) (Germany), BHPS/UKHLS (1996–2020) (UK).

Table 2 shows differences in life satisfaction and mental health between mothers who re-partnered within five years of the separation and mothers who remained single. Although the scores of re-partnered mothers in both Germany and the UK were slightly higher even before the separation, the differences were only significant from one year after entry into singlehood onward. For mental health, the scores of re-partnered mothers in Germany were also significantly higher than those of mothers who remained single only after entry into singlehood. For mothers in the UK, there were no significant mean differences in mental health at any time point. Mothers who did not re-partner were significantly older when they entered singlehood than mothers who re-partnered in both countries, indicating a lower likelihood of re-partnering for older single mothers. Similar patterns can be observed for the age at first birth and the age of the youngest child at entry into singlehood. In addition, mothers in the UK who remained single had significantly more children at entry into singlehood than those who re-partnered. In Germany, the proportion of mothers with high education was slightly higher among those who did not re-partner than among those who did. In the UK, there were no noticeable differences by education.

**Table 2.** Mean differences by re-partnering status.

	Germany			UK		
	Not re-partnered^a^	Re-partnered^1^	Diff.^2^	Not re-partnered^1^	Re-partnered^1^	Diff.^2^
Mean life satisfaction (measured at several distances from entry into singlehood)						
−2/-1 years	6.5	6.6		6.2	6.4	†
0 years	6.3	6.4		5.8	6.1	
+1/+2 years	6.5	6.9	***	6.0	6.6	***
+3/+5 years	6.6	7.1	***	6.0	6.9	***
Mean mental health (measured at several distances from entry into singlehood)						
−2/-1 years	46.2	45.1		45.9	45.9	
0 years	43.7	43.8		42.5	42.3	
+1/+2 years	45.9	47.4	*	44.6	45.3	
+3/+5 years	46.0	48.2	**	44.9	45.3	
Mean age at entry into singlehood	36.5	32.3	***	35.8	31.1	***
Mean age at first birth	25.1	23.8	***	25.9	24.7	***
Mean number of children at entry into singlehood	1.8	1.8		1.9	1.7	**
Mean age of youngest child at entry into singlehood	7.1	5.1	***	6.0	3.8	***
% of individuals with higher education at entry into singlehood	22.1	16.9	†	29.9	29.6	
N (individuals)	1214	480		820	250	
N (observations)	6262	3007		3830	1397	

*Source*: SOEP (1984–2020) (Germany), BHPS/UKHLS (1996–2020) (UK).

*Notes:* ^a^within 5 years of entry into singlehood; ^2^mean differences between groups derived from t-test results (^†^p < 0.1, *p < 0.05, **p < 0.01, ***p < 0.001).

### Multivariate results

**Figure 1.**
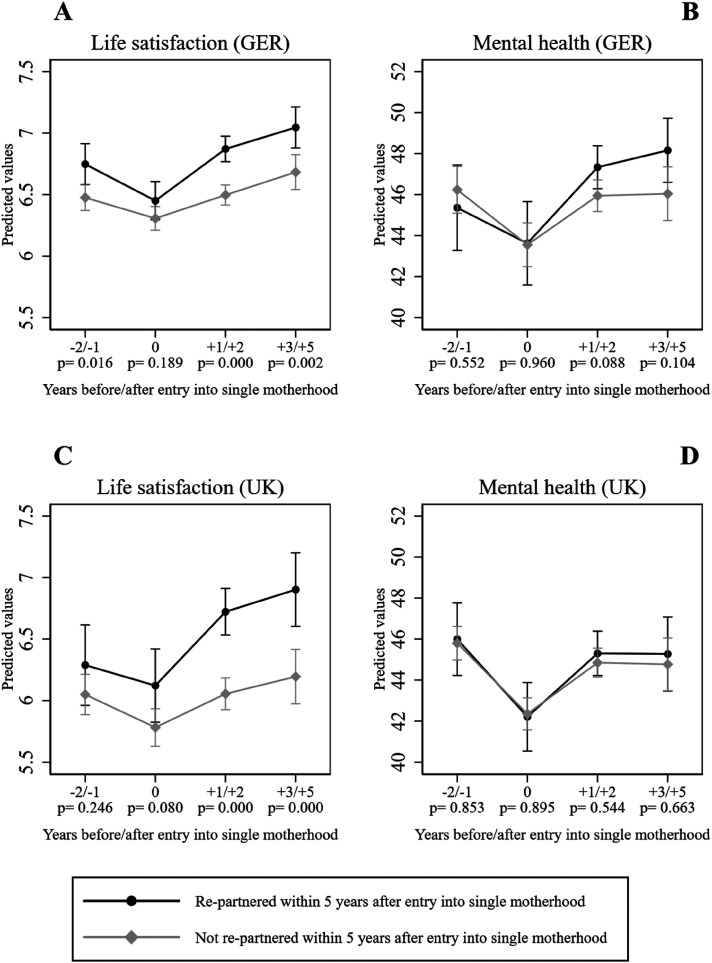
Predicted values of life satisfaction and mental health before and after entry into single motherhood (p-values under the x-axis are for the test between re-partnered and not re-partnered mothers).

Figure 1 shows the predicted life satisfaction and mental health trajectories before and after entry into single motherhood. The p-values under the x-axis indicate whether the differences between coefficients for each time point were significant between re-partnered and stably single mothers. Prior to their entry into single motherhood, re-partnered mothers in Germany (Figure 1A) had significantly higher levels of life satisfaction than stably single mothers. More substantial differences in the predicted life satisfaction of re-partnered and stably single mothers are found from one year after entry into singlehood until up to five years after, with the differences being highly significant. Although the decrease in life satisfaction potentially attributable to entering single motherhood was followed by an increase in both groups in subsequent years, the increase was steeper for re-partnered mothers. This indicates that re-partnering was positively associated with life satisfaction among German mothers.

For mental health in Germany (Figure 1B), we find that although the differences between stably single and re-partnered mothers before they entered singlehood were minor (p = 0.552), the drop in the year of the transition was less strong for those who re-partnered. We observe that for both groups, mental health improved in the two years after they entered single motherhood, with the improvement being slightly greater for the re-partnered group. Up to three to five years after entry into single motherhood, mental health is found to have stabilized for both groups, with no significant differences (p = 0.104).

Figure 1C shows that among British mothers, those who re-partnered had better life satisfaction prior to entering single motherhood and in the year of entry. Especially in the year of separation, the difference between the two groups was similar to that in the German context, but was not significant. After a decline in the year of separation, both the re-partnered and the stably singles experienced an increase in life satisfaction, but the increase was steeper for those who re-partnered. One to two years after entry into single motherhood, re-partnered mothers had significantly higher life satisfaction than mothers who remained single, while three to five years after entry into single motherhood, re-partnered mothers still had significantly higher life satisfaction.

In the UK, the mental health of mothers who re-partnered and of mothers who remained single did not differ substantially at any time point (Figure 1D). In addition, the differences in the trajectories of the two groups were smaller in the UK than in Germany, with p-values of differences ranging between 0.544 and 0.895. The mental health of both the re-partnered and the stably single mothers was higher in the two years after entry into single motherhood than in the year of entry.

## Discussion

In this paper, we used panel data from Germany and the UK to examine the extent to which differences in mental health and life satisfaction between re-partnered and stably single mothers were due to selection. We went beyond previous studies that considered health-related selection in the context of re-partnering among single mothers, but either did not use panel data (Recksiedler and Bernardi, 2019) or panel regression methods (Pevalin and Ermisch, 2004), or focused on only one country context and outcome (Kühn et al., 2023).

We showed that a higher share of single mothers re-partnered within five years of separation in Germany than in the UK. Older single mothers had a lower likelihood of re-partnering in both countries, highlighting the importance of timing, a key element in the life course perspective (Elder, 1994). The fixed effects analyses revealed two key findings: first, the trajectories of the two outcomes differed. Clear patterns showed that mothers who re-partnered within five years had higher life satisfaction scores even before becoming single mothers, but this difference was significant only in Germany. The differences widened further after entry into single motherhood, consistent with the findings of Kühn et al. (2023). This suggests that selection played a role in the differences, but was not the main driver. The life satisfaction benefits of re-partnering reflect both selection and causation processes (see Carr and Springer, 2010). For mental health, we found neither patterns of selection nor evidence of causation processes. These findings demonstrate the importance of examining multiple well-being dimensions when studying the re-partnering of single mothers, particularly when considering selection processes. The weak selection influence, especially for mental health, aligns with prior findings on the positive effects of re-partnering (see Gloor et al., 2021; Lin et al., 2019). Accordingly, we argue that more research on the drivers behind the re-partnering's impact on life satisfaction and mental health is needed.

Second, the relationship between re-partnering and life satisfaction was found to be more long-lasting in Germany, where re-partnered mothers still had significantly better scores five years after entry into single motherhood. Before they entered single motherhood, the differences between those who re-partnered and those who remained single were similar in both countries, but were significantly different only in Germany. This finding weakly supports Hypothesis 1, which anticipated that selection into re-partnering by well-being would be stronger in Germany than in the UK. The mental health patterns of the mothers who re-partnered differed between the two countries. While the increase in mental health was larger for mothers who re-partnered than for mothers who remained single in Germany, this was not the case in the UK, which could be explained by the stronger support for single mothers in Germany. Thus, mothers in Germany might have felt less pressure to find a new partner, potentially resulting in higher quality relationships.

One limitation that warrants further attention is that we did not distinguish between re-partnering events involving co-residence with a new partner and reconciliation with a former partner, despite previous research highlighting the importance of the latter (Nepomnyaschy and Teitler, 2013). Mothers who experience poor mental health and life satisfaction during single motherhood may be more likely to return to a former partner. Due to the lack of information in the BHPS/UKHLS data on partner identification numbers for unmarried partners, we were unable to account for this possibility in our analyses. Including this distinction in future analyses would contribute to a fuller understanding of the dynamics of re-partnering. Due to data limitations, we also disregarded non-co-resident partners. While the importance of these partners in re-partnering research has been pointed out by Bastin (2019), they are not sufficiently covered in the SOEP and UKHLS/BHPS data. Therefore, our approach did not enable us to differentiate between being in a romantic relationship and sharing a household with a new partner. Furthermore, the data do not include information on whether the separation was initiated by the mother. Including this information in future research on single mothers’ re-partnering behavior could provide important insights into the role of agency, an important element in the life course perspective (Elder, 1994), in single mothers’ well-being trajectories following a separation.

Our design clearly showed that in the five-year period after entry into singlehood, the life satisfaction (and mental health in Germany) of women who re-partnered was significantly better compared to women who remained single. However, as the exact timing of re-partnering was unclear, some of the women might have felt relief after the separation, which increased their life satisfaction and made them more open to re-partnering. Whether this was the case could not be clearly identified in our design. However, one indication that re-partnering did indeed increase life satisfaction was that a large proportion of the women re-partnered relatively quickly after the separation (see Table 1). Moreover, we found both the mental health and the life satisfaction of the women decreased in the year of separation and increased steadily thereafter. Another limitation of our research is that the number of single mothers tends to increase over time, which could make this group more heterogeneous, potentially leading to changes in the patterns of selection into re-partnering. Although the calendar year controls in our models could adjust for potential confounding, in future research, effect heterogeneity can only be investigated with moderation models based on larger sample sizes. An important potential source of heterogeneity in terms of well-being within the group of single mothers is the level of conflict and the relationship quality in the previous partnership. The well-being trajectories of mothers transitioning out of a relationship with particularly low relationship quality could be substantially different from those of other mothers, as they might show a less pronounced decline in well-being following the separation. Modeling this was not feasible with the underlying data, but future research should consider these factors.

Overall, we found indications of positive selection in re-partnering relationships with respect to life satisfaction in both Germany and the UK. Given the well-being disadvantages of single mothers and the potential benefits of re-partnering, these results provide important insights into a potential cumulative disadvantage of single mothers over the life course, particularly with regard to life satisfaction. Considering two similar but distinct outcomes was instructive, as we did not find comparable patterns for mental health. These results show that selection contributed to differences in life satisfaction between re-partnered and continuously single mothers, but did not fully explain the differences. Therefore, it would be informative for future research to further investigate the actual effects of the re-partnering transition.

## Supplemental Material

sj-docx-1-asj-10.1177_00016993241300435 - Supplemental material for Selective re-partnering? Mental health and life satisfaction among separated single mothers in Germany and the UK Supplemental material, sj-docx-1-asj-10.1177_00016993241300435 for Selective re-partnering? Mental health and life satisfaction among separated single mothers in Germany and the UK by Philipp Dierker, Mine Kühn and Mikko Myrskylä in Acta Sociologica
