# Supplementary material for: Selective re-partnering? Mental health and life satisfaction among separated single mothers in Germany and the UK
Source: Acta Sociol. 2025 Jan 8;68(3):357–69. doi: 10.1177/00016993241300435 (PMC12169628; doi:10.1177/00016993241300435)
Supplement: sj-docx-1-asj-10.1177_00016993241300435 - Supplemental material for Selective re-partnering? Mental health and life satisfaction among separated single mothers in Germany and the UK [file sj-docx-1-asj-10.1177_00016993241300435.docx]

**Selective re-partnering? Mental health and life satisfaction among separated single mothers in Germany and the UK**

Online Appendix

Contents

[1 Distributions 2](#_Toc168047289)

[2 Coding of education variables 3](#_Toc168047290)

[3 Different specifications of mother’s age as control variable 3](#_Toc168047291)

[4 Coefficients from fixed effects models 4](#_Toc168047292)

[5 SF-12 (Mental Component Summary Scale) 5](#_Toc168047293)

# Distributions


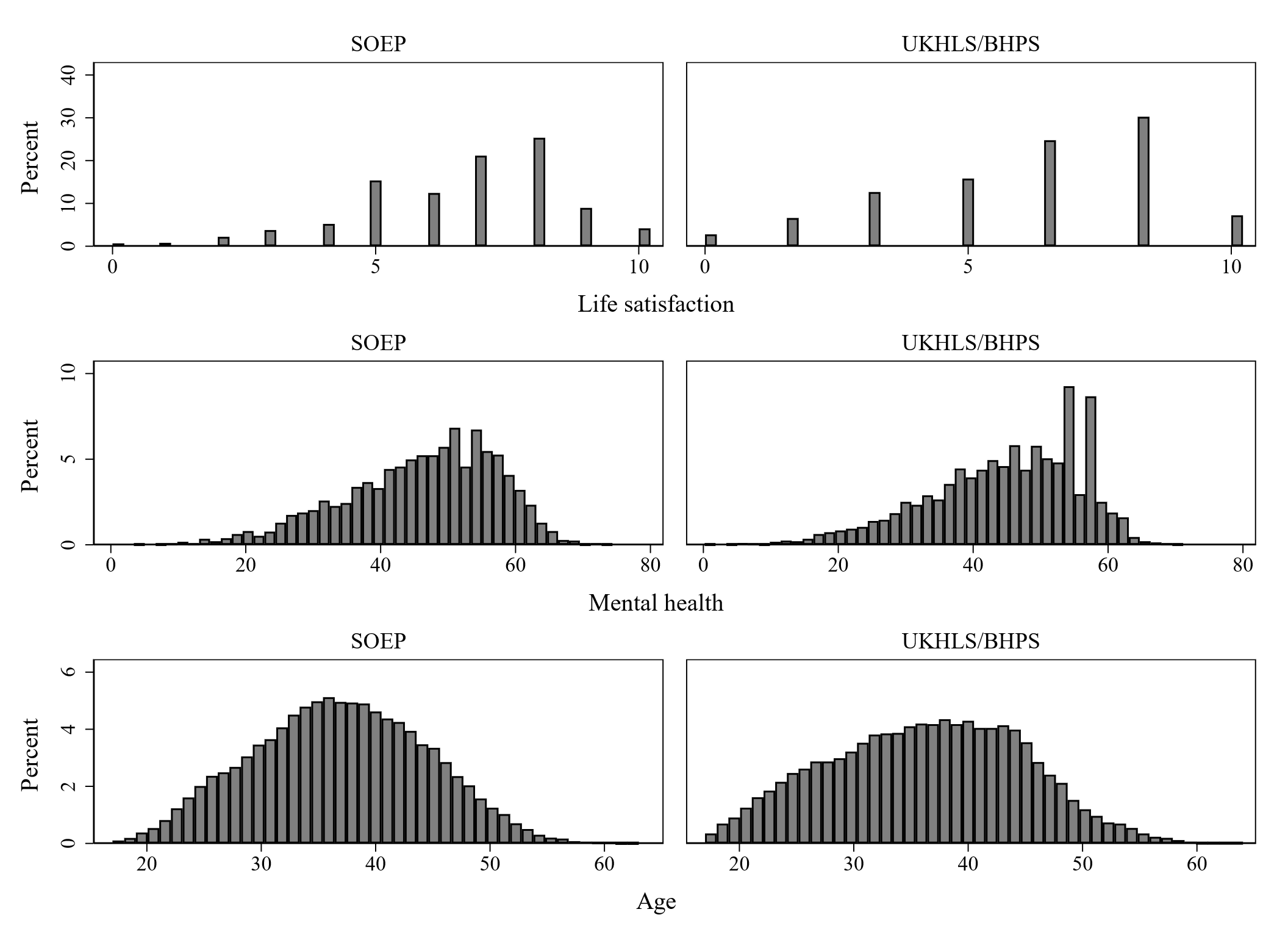


Figure A 1: Distributions of outcomes and age

# Coding of education variables

In the case of the SOEP dataset, educational levels “Doctoral or equivalent level,” “Master’s or equivalent level,” “Bachelor’s or equivalent level,” and “Short-cycle tertiary education” were classified as “high educational level,” while “Primary education,” “Lower secondary education,” “Upper secondary education,” and “Post-secondary non-tertiary education” were classified as “low educational level”. In the UKHLS/BHPS dataset, categories “college of further education,” “polytechnic,” and “university” were categorized as “high education level,” while “nursing school,” “other training establishment,” and “none of the above” were classified as “low educational level”.

# Different specifications of mother’s age as control variable

Table A 1: AIC-values (best fit in grey)

|  | GER | | | | UK | | | |
| --- | --- | --- | --- | --- | --- | --- | --- | --- |
|  | Life satisfaction | | Mental health | | Life satisfaction | | Mental health | |
|  | Not re-partnered | Re-partnered | Not re-partnered | Re-partnered | Not re-partnered | Re-partnered | Not re-partnered | Re-partnered |
| 1-year dummies | 21818.59 | 10493.09 | **15134.65** | 5646.204 | 14855.14 | 5547.546 | 17643.1 | 4951.83 |
| 2-year dummies | **21790.15** | 10472.05 | 15154.46 | 5633.754 | 14844.8 | 5520.752 | 17637.41 | 4925.978 |
| 3-year dummies | 21794.76 | 10473.83 | 15165.32 | 5642.08 | 14838.87 | 5517.35 | 17635.84 | 4918.635 |
| 4-year dummies | 21804.44 | **10462.17** | 15142.31 | **5631.046** | 14835.65 | **5508.83** | 17632.8 | 4915.406 |
| 5-year dummies | 21814.02 | 10462.63 | 15144.49 | 5638.45 | **14828.72** | 5513.745 | **17630.6** | **4914.711** |

Table A 2: BIC-values (best fit in grey)

|  | GER | | | | UK | | | |
| --- | --- | --- | --- | --- | --- | --- | --- | --- |
|  | Life satisfaction | | Mental health | | Life satisfaction | | Mental health | |
|  | Not re-partnered | Re-partnered | Not re-partnered | Re-partnered | Not re-partnered | Re-partnered | Not re-partnered | Re-partnered |
| 1-year dummies | 22148.85 | 10775.47 | 15398.18 | 5831.86 | 15173.38 | 5783.278 | 17918.15 | 5148.798 |
| 2-year dummies | 21972.13 | 10628.26 | 15303.41 | 5738.483 | 15019.52 | 5651.715 | 17789.56 | 5035.913 |
| 3-year dummies | 21929.56 | 10587.98 | 15279.89 | 5723.007 | 14963.66 | 5611.643 | 17747.02 | 4996.506 |
| 4-year dummies | 21912.28 | 10558.3 | 15228.25 | **5692.932** | 14935.49 | 5587.408 | 17720.58 | 4984.116 |
| 5-year dummies | **21908.38** | **10540.73** | **15224.7** | 5695.575 | **14916.07** | **5581.846** | **17706.68** | **4974.26** |

# Coefficients from fixed effects models

Table A 3: Coefficients of comparison between life satisfaction and mental health of non-re-partnered and re-partnered mothers

|  | (1) | (2) | (3) | (4) | (5) | (6) | (7) | (8) |
| --- | --- | --- | --- | --- | --- | --- | --- | --- |
|  | Life satisfaction | Life satisfaction | Life satisfaction | Life satisfaction | SF-12 Mental Component Summary (PCS) | SF-12 Mental Component Summary (PCS) | SF-12 Mental Component Summary (PCS) | SF-12 Mental Component Summary (PCS) |
|  | b/se | b/se | b/se | b/se | b/se | b/se | b/se | b/se |
| -2/-1 |  |  |  |  |  |  |  |  |
|  |  |  |  |  |  |  |  |  |
| 0 | -0.17^**^ | -0.30^**^ | -0.27^**^ | -0.17 | -2.68^***^ | -1.74 | -3.45^***^ | -3.78^***^ |
|  | (0.06) | (0.10) | (0.10) | (0.18) | (0.71) | (1.18) | (0.51) | (1.03) |
| +1/+2 | 0.02 | 0.12 | 0.01 | 0.43^*^ | -0.29 | 1.98^+^ | -0.95 | -0.69 |
|  | (0.08) | (0.11) | (0.12) | (0.20) | (0.77) | (1.20) | (0.62) | (1.10) |
| +3/+5 | 0.21^+^ | 0.30^+^ | 0.15 | 0.61^*^ | -0.19 | 2.80^+^ | -1.03 | -0.72 |
|  | (0.11) | (0.15) | (0.18) | (0.29) | (1.12) | (1.68) | (0.96) | (1.63) |
| high_educ=0 | 0.00 | 0.00 | 0.00 | 0.00 | 0.00 | 0.00 | 0.00 | 0.00 |
|  | (.) | (.) | (.) | (.) | (.) | (.) | (.) | (.) |
| high_educ=1 | 0.49 | -0.14 | -0.43 | -0.05 | 1.59 | 8.05 | -0.32 | 1.30 |
|  | (0.41) | (0.34) | (0.46) | (0.56) | (5.43) | (5.80) | (2.34) | (2.81) |
| c5age=16 | 0.00 | 0.00 | 0.00 | 0.00 | 0.00 | 0.00 | 0.00 | 0.00 |
|  | (.) | (.) | (.) | (.) | (.) | (.) | (.) | (.) |
| c5age=21 | -0.57 | -0.99^*^ | -0.49 | -0.88^+^ | -0.30 | -0.43 | -2.85 | 0.90 |
|  | (0.40) | (0.40) | (0.39) | (0.46) | (4.76) | (5.53) | (2.26) | (4.76) |
| c5age=26 | -1.01^*^ | -1.05^*^ | -0.68 | -0.99^+^ | -1.04 | 1.25 | -5.60^*^ | 0.55 |
|  | (0.44) | (0.44) | (0.48) | (0.58) | (5.20) | (5.78) | (2.74) | (5.14) |
| c5age=31 | -1.26^**^ | -1.08^*^ | -1.31^*^ | -1.27^+^ | -1.47 | 0.69 | -9.19^**^ | -1.80 |
|  | (0.47) | (0.48) | (0.55) | (0.69) | (5.46) | (6.24) | (3.11) | (5.55) |
| c5age=36 | -1.21^*^ | -1.06^*^ | -1.55^*^ | -1.84^*^ | -1.57 | -2.75 | -11.15^**^ | -3.09 |
|  | (0.49) | (0.53) | (0.61) | (0.82) | (5.71) | (6.68) | (3.42) | (6.09) |
| c5age=41 | -1.19^*^ | -0.86 | -1.77^**^ | -1.81^+^ | 0.17 | -2.33 | -12.18^**^ | -2.27 |
|  | (0.52) | (0.58) | (0.67) | (0.96) | (6.01) | (7.15) | (3.75) | (6.71) |
| c5age=46 | -1.39^*^ | -0.80 | -1.66^*^ | -1.88^+^ | 0.88 | -1.54 | -11.29^**^ | -2.97 |
|  | (0.56) | (0.66) | (0.75) | (1.13) | (6.31) | (7.82) | (4.12) | (7.45) |
| c5age=51 | -1.25^*^ | 0.07 | -2.16^*^ | -2.76^*^ | 2.77 | -0.78 | -12.89^**^ | -7.23 |
|  | (0.61) | (0.83) | (0.85) | (1.36) | (6.75) | (9.50) | (4.64) | (8.39) |
| c5age=56 | -0.97 | 2.02 | -1.71 | -3.35 | 17.31^*^ |  | -12.25^*^ | -5.05 |
|  | (0.79) | (1.42) | (1.05) | (2.12) | (8.21) |  | (5.60) | (10.88) |
| c5age=61 | -1.83 |  | -1.83 |  | 9.78 |  |  |  |
|  | (2.06) |  | (1.93) |  | (15.09) |  |  |  |
| c5period=1984 |  |  |  |  |  |  |  |  |
|  |  |  |  |  |  |  |  |  |
| c5period=1989 | 0.06 | 0.77^**^ |  |  |  |  |  |  |
|  | (0.20) | (0.26) |  |  |  |  |  |  |
| c5period=1994 | 0.33 | 1.15^***^ |  |  |  |  |  |  |
|  | (0.26) | (0.33) |  |  |  |  |  |  |
| c5period=1999 | 0.27 | 1.32^***^ | 0.21 | 0.34 |  |  |  |  |
|  | (0.33) | (0.40) | (0.24) | (0.31) |  |  |  |  |
| c5period=2004 | -0.08 | 1.41^**^ | 0.14 | 0.17 | -0.33 | 1.87 |  |  |
|  | (0.37) | (0.47) | (0.32) | (0.43) | (1.18) | (1.51) |  |  |
| c5period=2009 | 0.07 | 1.50^**^ | 0.06 | -0.23 | -1.65 | 1.62 |  |  |
|  | (0.43) | (0.54) | (0.26) | (0.55) | (1.79) | (2.37) |  |  |
| c5period=2014 | 0.06 | 1.60^**^ | 0.10 | -0.09 | 0.94 | 5.73^+^ | 1.69^*^ | 1.00 |
|  | (0.47) | (0.61) | (0.20) | (0.48) | (2.41) | (3.26) | (0.66) | (1.09) |
| c5period=2019 | 0.00 | 1.66^*^ | 0.00 | 0.00 | 0.81 | 6.96^+^ | 2.12^+^ | 2.74 |
|  | (0.51) | (0.68) | (.) | (.) | (2.85) | (4.14) | (1.26) | (2.47) |
| Constant | 7.47^***^ | 6.45^***^ | 7.48^***^ | 7.64^***^ | 45.98^***^ | 41.31^***^ | 54.81^***^ | 46.48^***^ |
|  | (0.57) | (0.58) | (0.58) | (0.67) | (5.78) | (6.23) | (3.17) | (5.42) |
| N (observations) | 6248 | 3005 | 3789 | 1392 | 2273 | 863 | 2571 | 721 |
| N (individuals) | 1213 | 480 | 815 | 250 | 945 | 343 | 574 | 122 |

# SF-12 (Mental Component Summary Scale)

Table A 4: Questions and options for answers from which SF-12 scales are derived

| **Variable** | **Question** | **Options** |
| --- | --- | --- |
| General health | “In general, would you say your health is…” | 1 (“Excellent”), 2 (“Very good”), 3 (“Good”), 4 (“Fair”), 5 (“Poor”) |
| Health limits moderate activities | “The following questions are about activities you might do during a typical day. Does your health now limit you in these activities? If so, how much? Moderate activities, such as moving a table, pushing a vacuum cleaner, bowling or playing golf” | 1 (“Yes, limited a lot”), 3 (“Yes, limited a little”), 3 (“No, not limited at all”) |
| Health limits several flights of stairs | “The following questions are about activities you might do during a typical day. Does your health now limit you in these activities? If so, how much? Climbing several flights of stairs” | 1 (“Yes, limited a lot”), 3 (“Yes, limited a little”), 3 (“No, not limited at all”) |
| Last 4 weeks: Physical health limits amount of work | “During the past 4 weeks, how much of the time have you had any of the following problems with your work or other regular daily activities as a result of your physical health? Accomplished less than you would like” | 1 (“All of the time”), 2 (“Most of the time”), 3 (“Some of the time”), 4 (“A little of the time”), 5 (“None of the time”) |
| Last 4 weeks: Physical health limits kind of work | “During the past 4 weeks, how much of the time have you had any of the following problems with your work or other regular daily activities as a result of your physical health? Were limited in the kind of work or other activities” | 1 (“All of the time”), 2 (“Most of the time”), 3 (“Some of the time”), 4 (“A little of the time”), 5 (“None of the time”) |
| Last 4 weeks: Mental health meant accomplished less | “During the past 4 weeks, how much of the time have you had any of the following problems with your work or other regular daily activities as a result of any emotional problems (such as feeling depressed or anxious)? Accomplished less than you would like” | 1 (“All of the time”), 2 (“Most of the time”), 3 (“Some of the time”), 4 (“A little of the time”), 5 (“None of the time”) |
| Last 4 weeks: Mental health meant worked less carefully | “During the past 4 weeks, how much of the time have you had any of the following problems with your work or other regular daily activities as a result of any emotional problems (such as feeling depressed or anxious)? Did work or other activities less carefully than usual” | 1 (“All of the time”), 2 (“Most of the time”), 3 (“Some of the time”), 4 (“A little of the time”), 5 (“None of the time”) |
| Last 4 weeks: Pain interfered with work | “During the past 4 weeks, how much did pain interfere with your normal work (including both work outside the home and housework)?” | 1 (“Not at all”), 2 (“A little bit”), 3 (“Moderately”), 4 (“Quite a bit”), 5 (“Extremely”) |
| Last 4 weeks: Felt calm and peaceful | “These questions are about how you feel and how things have been with you during the past 4 weeks. For each question, please give the one answer that comes closest to the way you have been feeling. How much of the time during the past 4 weeks… Have you felt calm and peaceful?” | 1 (“All of the time”), 2 (“Most of the time”), 3 (“Some of the time”), 4 (“A little of the time”), 5 (“None of the time”) |
| Last 4 weeks: Had a lot of energy | “These questions are about how you feel and how things have been with you during the past 4 weeks. For each question, please give the one answer that comes closest to the way you have been feeling. How much of the time during the past 4 weeks… Did you have a lot of energy?” | 1 (“All of the time”), 2 (“Most of the time”), 3 (“Some of the time”), 4 (“A little of the time”), 5 (“None of the time”) |
| Last 4 weeks: Felt downhearted and depressed | “These questions are about how you feel and how things have been with you during the past 4 weeks. For each question, please give the one answer that comes closest to the way you have been feeling. How much of the time during the past 4 weeks… Have you felt downhearted and depressed?” | 1 (“All of the time”), 2 (“Most of the time”), 3 (“Some of the time”), 4 (“A little of the time”), 5 (“None of the time”) |
| Last 4 weeks: Physical or mental health interfered with social life | During the past 4 weeks, how much of the time has your physical health or emotional problems interfered with your social activities (like visiting friends, relatives)?” | 1 (“All of the time”), 2 (“Most of the time”), 3 (“Some of the time”), 4 (“A little of the time”), 5 (“None of the time”) |
